# Real-world effectiveness of aromatase inhibitors and fulvestrant in HR+/HER2- advanced breast cancer: a snapshot of the last two years before conventional use of CDK 4/6 inhibitors in a Portuguese institution

**DOI:** 10.1080/20523211.2023.2296551

**Published:** 2024-01-17

**Authors:** Maria Inês Teodoro, Alexandra Mayer, Ana da Costa Miranda, Hugo Nunes, Filipa Alves da Costa, António Lourenço

**Affiliations:** a Unidade de Investigação em Epidemiologia, Instituto Português de Oncologia de Lisboa Francisco Gentil, E.P.E., Lisbon, Portugal; b Research Institute for Medicines (iMED), Faculty of Pharmacy, University of Lisbon, Lisbon, Portugal; c Egas Moniz Center for Interdisciplinary Research (CiiEM), Egas Moniz School of Health and Science, Almada, Portugal; d Medical Oncology Department, Instituto Português de Oncologia de Lisboa Francisco Gentil, E.P.E., Lisbon, Portugal; e NOVA Medical School, Universidade Nova de Lisboa, Lisboa, Portugal

**Keywords:** Advanced breast cancer, Registries, Effectiveness, Fulvestrant, Aromatase inhibitors

## Abstract

**Background:**

Monotherapy with aromatase inhibitors and fulvestrant were the standard-of-care for hormone receptor-positive (HR+)/human epidermal growth factor receptor-type2 negative (HER2-) advanced breast cancer, before integration of cyclin-dependent kinase 4/6 inhibitors. Effectiveness data is essential for regulatory action, but little is known about real-world use of aromatase inhibitors and fulvestrant.

**Methods:**

A retrospective cohort study was conducted resorting to data from a cancer registry to identify adult women with HR+/HER- advanced breast cancer exposed to aromatase inhibitors or fulvestrant (31 May 2017–31 March 2019) at the main oncology hospital in Portugal. Cases were updated with follow-up until death or cut-off (31 March 2021) and pseudoanonymized data extracted. Primary outcome was overall survival (OS) and secondary time to treatment failure (TTF), estimated using survival analysis and compared with published trials.

**Results:**

192 patients were distributed by subgroups according to the medicine. Letrozole: OS 30.8 (95% confidence interval (CI) 20.6–41.4); TTF 11.2 (95%CI 8.7–13.7). Exemestane: OS 22.1 (95%CI 9.7–34.6); TTF 6.0 (95%CI 4.1–7.8). Fulvestrant: OS 21.6 (95%CI 16.5–26.7); TTF 5.6 (95%CI 4.5–6.6).

**Conclusions:**

Estimated effectiveness (OS) of letrozole and fulvestrant was, respectively, 3.2–3.5 months lower than reported. The clinical meaning seems uncertain and may be explained a higher proportion of worse prognostic characteristics in patients treated in the real-world.

## 1. Introduction

Worldwide, breast cancer is the most commonly diagnosed cancer (World Health Organization, 2020). Although 90–95% of breast cancers are diagnosed in early stages, there are still many women who do not have access to screening, leading to diagnosis at advanced stages (Akram et al., 2017).

According to the European Society of Medical Oncology, advanced breast cancer (ABC) comprises inoperable and metastatic breast cancer (stages IIIC and IV, respectively) and is the leading cause of mortality by cancer among women. ABC has a median overall survival (OS) of approximately 3 years and a 5-year survival rate of 25% (Cardoso et al., 2020; Gobbini et al., 2018; Howlader et al., 2016). As ABC cannot be cured, therapeutic goals include palliation of symptoms, improving quality of life and increasing survival. Endocrine therapy (ET) is recommended as first-line treatment for hormone receptor-positive (HR+)/human epidermal growth factor receptor-type 2 negative (HER2-) ABC, considering its demonstrated benefits in terms of increased progression-free survival (PFS) and time to progression (TTP), decreased mortality and reduced toxicity (Cardoso et al., 2020; Cheung, 2007). ET comprises three main therapeutic options that differ in mechanism of action: tamoxifen, aromatase inhibitors (AIs – letrozole, exemestane and anastrozole) and fulvestrant. For over two decades, tamoxifen was considered first-line therapy in ABC eligible for ET, but it has been replaced by AIs and fulvestrant due to their superior efficacy and tolerability (Gluck et al., 2002; Mouridsen et al., 2003; Zhang et al., 2017).

Currently, cyclin-dependent kinase 4/6 inhibitors (CDK4/6i) (palbociclib, ribociclib or abemaciclib) in combination with an AI or fulvestrant are the standard-of-care in HR+/HER2- ABC (Battisti et al., 2018; Cardoso et al., 2020; Ding et al., 2018). However, there are still several patients who, due to their comorbidities or to avoid toxicities inherent to CDK4/6i, can undergo monotherapy with AI or fulvestrant (McAndrew & Finn, 2020).

Despite the existence of real-world studies about the effectiveness of letrozole and fulvestrant in the abovementioned indications, these studies are outdated and have methodological limitations, suggesting that additional studies can contribute to more realistic expectations (Liu et al., 2013; Martínez Marín et al., 2009; Steger et al., 2005; Yoo et al., 2011). Steger et al. led a unicentric retrospective cohort study including 126 patients treated with fulvestrant after previous ET, with median follow-up of 13.0 months, reporting an overall response rate (ORR) of 9.5%, and clinical benefit (CB) in 43.6% of patients (2005). The study by Martínez Marín et al included 36 patients treated with fulvestrant, with a median follow-up of 8.9 months (2009), reporting a TTP was 4.2 months (95%CI 2.6–5.8), and 31.4% of patients achieving CB. The study by Yoo et al. was even smaller, with only 19 patients exposed to fulvestrant. With a median follow-up of 7.4 months, the median TTP reported was 5.5 months (95%CI 0.4–10.7) and median OS of 17.9 months (95%CI 2.7–33.1) (2011). Finally, Liu et al. reported a study including 35 premenopausal women treated with letrozole, with a median follow-up of 44.0 months and estimated a median OS of 33.0 month and a PFS of 9.6 months (2013). This highlights a gap in updated and solid evidence about the effectiveness of such treatments.

In fact, recent studies also conducted in real-world contexts but focusing on the effectiveness of CDK4/6i in association with AI/fulvestrant have stressed that additional research on the effectiveness of ET in monotherapy is needed to better understand the true effectiveness of the combination (Alves da Costa et al., 2023; Cardoso Borges et al., 2022). This may be achieved by direct or indirect comparison of real-world effectiveness, resorting to registry data, with clinical trial efficacy outcomes, as described in various studies (Bjartell et al., 2021; Karim et al., 2018). Previous experience using the Portuguese Cancer Registry has demonstrated its potential value for pharmacoepidemiological and outcomes research studies, which are fundamental to providing information for clinical and health technology assessment decisions. While some of these previous studies have primarily focused safety issues (Aguiar et al., 2018), most have explored the efficacy-effectiveness gap with different treatments and for varied indications, including advanced melanoma (Borges et al., 2020), non-small cell lung cancer (Costa et al., 2019; Murteira et al., 2020) (Costa et al., 2019; Murteira et al., 2020) and advanced breast cancer (Alves da Costa et al., 2023; Cardoso Borges et al., 2022). Following these successful experiences, and aiming to overcome knowledge gaps, the current study aimed to evaluate the effectiveness of AIs and fulvestrant in HR+/HER2- ABC at the Portuguese Institute of Oncology of Lisbon (*Instituto Português de Oncologia de Lisboa Francisco Gentil*, IPOLFG).

## 2. Materials and methods

### 2.1. Study design

A retrospective cohort study, reported according to STROBE guidelines (von Elm et al., 2007), was conducted in which women with ABC who started therapy with AI or fulvestrant at IPOLFG between 31 May 2017 and 31 March 2019 and who met the eligibility criteria (described below) were included. The study period selected represents the last two years before CDK4/6i started to be conventionally used in HR+/HER2- ABC treatment at IPOLFG.

Cases were divided into four cohorts according to the medicine they were exposed to: letrozole, exemestane, anastrozole or fulvestrant. All patients who were treated with fulvestrant were included in the fulvestrant cohort, even if they were previously treated with AI. In cases that underwent therapy with more than one AI, the patient was included in the cohort of the AI administered first, as long as at least 2 cycles of treatment were administered. This approach was taken so that the focus was placed on first-line treatment of metastatic disease.

Patients were followed-up until death or cut-off date (31 May 2021). Estimated outcomes were compared with those published in the clinical trials (OS in the letrozole and fulvestrant cohorts) and time to treatment failure (TTF), in the letrozole and exemestane cohorts, and safety in all cohorts (Di Leo et al., 2010; Kaufmann et al., 2000; Mouridsen et al., 2003). Comparison of baseline characteristics at treatment initiation were also considered to interpret the efficacy-effectiveness gap.

### 2.2. Setting and data sources

This study used data from RON, a nationwide population-based cancer registry, which stores relevant information from cancer diagnosis until death. However, as stipulated by the International Agency for Research on Cancer, there is no full automatic migration of all data, contributing to higher quality but also to some delay in data update, as some variables require manual validation (International Agency for Research on Cancer, 1991). This implies that ahead of any effectiveness study, a process to ensure completeness and exhaustiveness is instituted by resorting to other sources of information. In this specific study, the IPOLFG Hospital Pharmacy database was used to identify all cases of interest. Subsequently, clinical records of patients were verified to assess eligibility criteria and updated in RON’s database (Assembleia da República, 2017). Finally, data were extracted in a pseudoanonymized format to perform the analysis.

### 2.3. Study population and information of interest

Women aged ≥18 years, with HR+ (oestrogen receptor + and/or progesterone receptor +)/HER2- ABC (regardless of metastasis’ location) and who underwent monotherapy (at least 2 cycles of treatment), at the IPOLFG, with an AI (in first-line) or fulvestrant (in first or second-line after disease progression with an AI), between 31 May 2017 and 31 March 2019, were included. There were no additional exclusion criteria. Information of interest included (a) demographic and clinical characteristics: age and stage at diagnosis, date of diagnosis, tumour location, histological type, HR status, and HER2 status and age, disease extension and Eastern Cooperative Oncology Group Performance Status (ECOG PS) at treatment initiation; (b) therapeutic characterisation variables: initiation date, date and reason for discontinuation (if applicable), adverse events (AEs) leading to treatment discontinuation (if applicable); and (c) outcomes and posttreatment characterisation: disease progression and date (if applicable), vital status and date of last known contact/death.

### 2.4. Study outcomes

The primary outcome was OS, defined as the time between treatment initiation and the date of death due to any cause. Secondary outcomes were TTF, defined as the time between treatment initiation and discontinuation due to any cause, and safety profile (proportion of patients who discontinued treatment due to AEs and its quantitative and qualitative description).

### 2.5. Statistical analysis

Prior to statistical analysis, the exhaustiveness of the data was evaluated by analysing the proportion of missing data for each variable. Data validity and accuracy were ensured by internal quality checks. After validation, data were exported from RON’s database to Microsoft Excel software, version 16.45. All statistical analyses were performed using IBM SPSS software, version 27.0. Demographic, clinical and therapeutic variables were characterised using descriptive statistics. Time-to-event outcomes (TTF and OS) were estimated using survival analysis through the Kaplan–Meier estimator. Median time to events and 1- and 2-year survival rates were reported using a 95% confidence interval (95%CI). Patients without the event of interest were censored at the cut-off date, and patients lost to follow-up were censored at the date of last contact. Thus, to estimate the TTF, patients who did not discontinue the medicine of interest were censored, and to estimate the OS, living patients were censored.

## 3. Results

**Figure 1.**
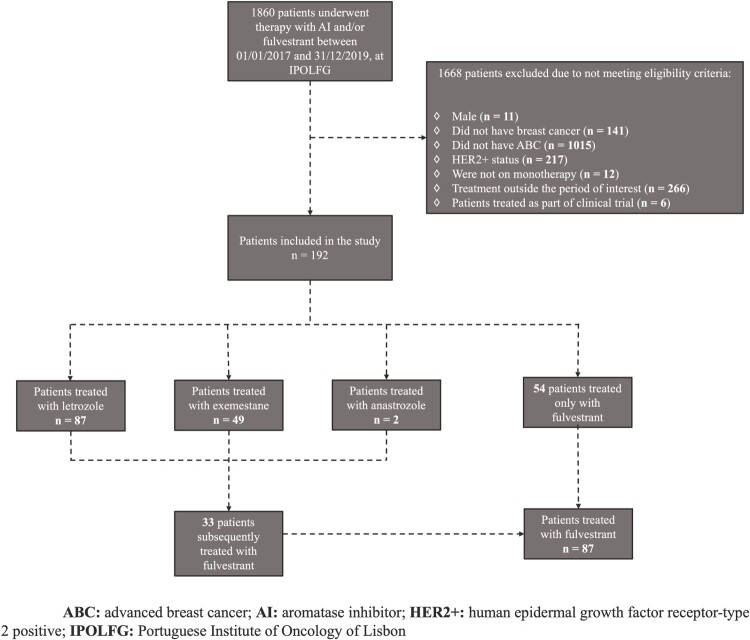
Flowchart of patient selection.

A total of 1860 cancer patients who were treated with AIs or fulvestrant were identified. Following the verification of eligibility criteria, 192 women (34 pre/perimenopausal and 157 postmenopausal) with HR+/HER2- ABC were selected and subsequently distributed into the four cohorts (Figure 1). Anastrozole analysis was dropped given its limited sample. Detailed demographic and clinical characteristics of included patients is provided in Table 1. A total of 4 patients (2.1%) were lost to follow-up (2 in the letrozole cohort, 1 in the fulvestrant cohort and 1 in both cohorts).

**Table 1.** Demographic and clinical characterisation of included patients.

Characteristics	* *	Letrozole cohort *n* = 87	Exemestane cohort *n* = 49	Fulvestrant cohort *n* = 87
Age at diagnosis, years	Median (IQR 25–75)	56 (47–74)	51 (42–61)	53 (45 –63)
	[Min.; Max.]	[29; 98]	[30; 82]	[28; 86]
Topography, n (%)	C50.1 – central portion of breast	6 (6.9)	4 (8.2)	5 (5.8)
	C50.2 – upper-inner quadrant of breast	1 (1.2)	2 (4.1)	5 (5.8)
	C50.3 – lower-inner quadrant of breast	4 (4.6)	1 (2.0)	4 (4.6)
	C50.4 – upper-outer quadrant of breast	22 (25.3)	8 (16.3)	15 (17.2)
	C50.5 – lower-outer quadrant of breast	1 (1.2)	3 (6.1)	4 (4.6)
	C50.8 – overlapping sites of breast	47 (54.0)	24 (49.0)	40 (46.0)
	C50.9 – breast NOS	6 (6.9)	7 (14.3)	14 (16.1)
Histological type, n (%)	M8500 – ductal carcinoma NOS	67 (77.0)	35 (71.4)	67 (77.0)
	M8507 – intraductal micropapillary carcinoma	1 (1.2)	1 (2.0)	0 (0.0)
	M8520 – lobular carcinoma NOS	5 (5.8)	6 (12.2)	5 (5.8)
	M8522 – infiltrating duct and lobular carcinoma	2 (2.3)	1 (2.0)	5 (5.8)
	M8523 – infiltrating duct mixed with other types of carcinoma	4 (4.6)	2 (4.2)	2 (2.3)
	M8524 – infiltrating lobular mixed with other types of carcinoma	1 (1.2)	1 (2.0)	1 (1.2)
	Other specified	4 (4.6)	0 (0.0)	4 (4.6)
	M8010 – carcinoma NOS	3 (3.5)	3 (6.1)	3 (3.5)
Stage at diagnosis, n (%)	IA	0 (0.0)	0 (0.0)	1 (1.2)
	IB	10 (11.5)	4 (8.2)	10 (11.5)
	IIA	13 (14.9)	14 (28.6)	15 (17.2)
	IIB	6 (6.9)	10 (20.4)	18 (20.7)
	IIIA	4 (4.6)	1 (2.0)	5 (5.8)
	IIIB	6 (6.9)	2 (4.2)	5 (5.8)
	IIIC	5 (5.8)	1 (2.0)	8 (9.2)
	IV	41 (47.1)	15 (30.6)	22 (25.3)
	Unknown	2 (2.3)	2 (4.2)	3 (3.5)

**ECOG PS:** Eastern Cooperative Oncology Group Performance Status; **IQR:** interquartile range; **Max:** maximum; **Min:** minimum; **NOS:** not otherwise specified.

### 3.1. Letrozole cohort

**Figure 2.**
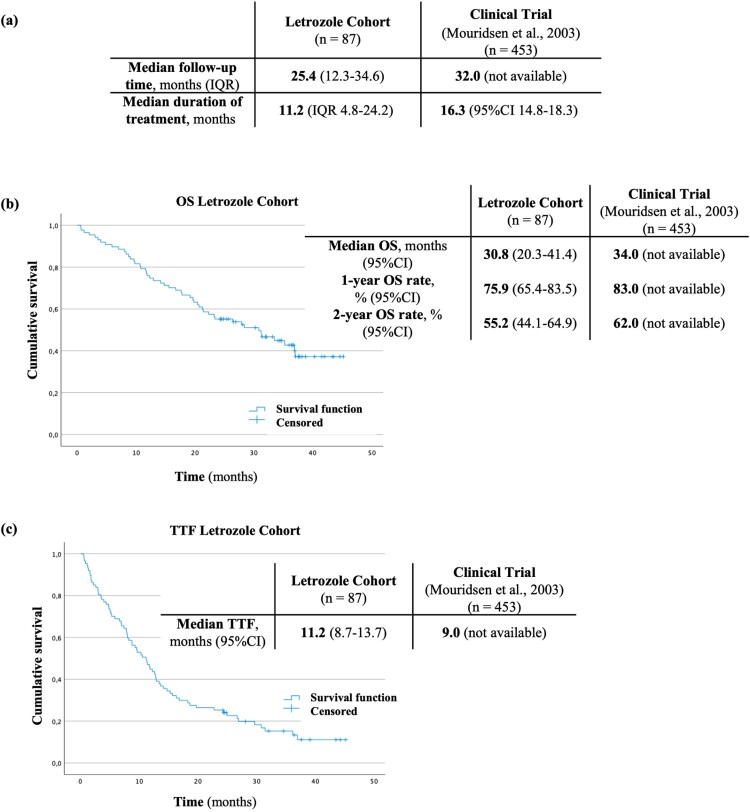
Median follow-up time (a), Kaplan–Meier estimate for OS (b) Kaplan–Meier estimate for TTF (c), in the letrozole cohort and the corresponding clinical trial.

All patients were treated with the recommended dosage, 2.5 mg/day. At treatment initiation, median age of patients was 64 years [interquartile range (IQR): 53–79], similar to reported in the clinical trial (65 years [IQR: 31–96]). Most patients (96.6%) had metastatic disease at letrozol initiation, as reported in the trial (93.6%) (Mouridsen et al., 2003). In both groups, ECOG PS 0 and 1 were the most frequent (37.9% in the letrozole cohort vs. 55.9% in the trial and 35.6% in the letrozole cohort vs. 37.5% in the trial) (Table 2). In the letrozole cohort, the median follow-up was 25.4 months (IQR: 12.3–34.6), and the median duration of treatment was 11.2 months (IQR: 4.8–24.2) (Figure 2(A)).

**Table 2.** Demographic and clinical characterisation of patients included in the letrozole cohort and in the respective clinical trial at treatment initiation.

		Letrozole cohort (*n* = 87)	Clinical trial (Mouridsen et al., 2003) (*n* = 453)
Age at treatment initiation, years	Median (IQR 25–75)	64 (53–79)	65 (31–96)
	[Min.; Max.]	[29; 98]	Not available
Disease extent at treatment initiation, n (%)	IIIC	3 (3.5)	29 (6.4)
	IV	84 (96.6)	422 (93.2)
ECOG PS at treatment initiation, n (%)	0	33 (37.9)	253 (55.8)
	1	31 (35.6)	170 (37.5)
	2	11 (12.6)	30 (6.2)
	3	5 (5.7)	–
	4	0 (0.0)	–
	Unknown	7 (8.1)	–

**IQR:** interquartile range; **ECOG PS:** Eastern Cooperative Oncology Group Performance Status.

Median OS was estimated to be 30.8 months (95%CI 20.3–41.4), and OS rates at 1- and 2-years were 75.9% (95%CI 65.4–83.5) and 55.2% (95%CI 44.1–64.9), respectively. Median TTF was estimated at 11.2 months (95%CI 8.7–13.7). The clinical trial estimated median OS and 1- and 2-year OS rates of 34.0 months, 83.0% and 62.0%, respectively, and a median TTF of 9.0 months (Mouridsen et al., 2003). Thus, the estimated values for the letrozole cohort were lower than those reported in the trial, in contrast to the TTF, which was higher in the real-world (Figure 2).

### 3.2. Exemestane cohort

**Figure 3.**
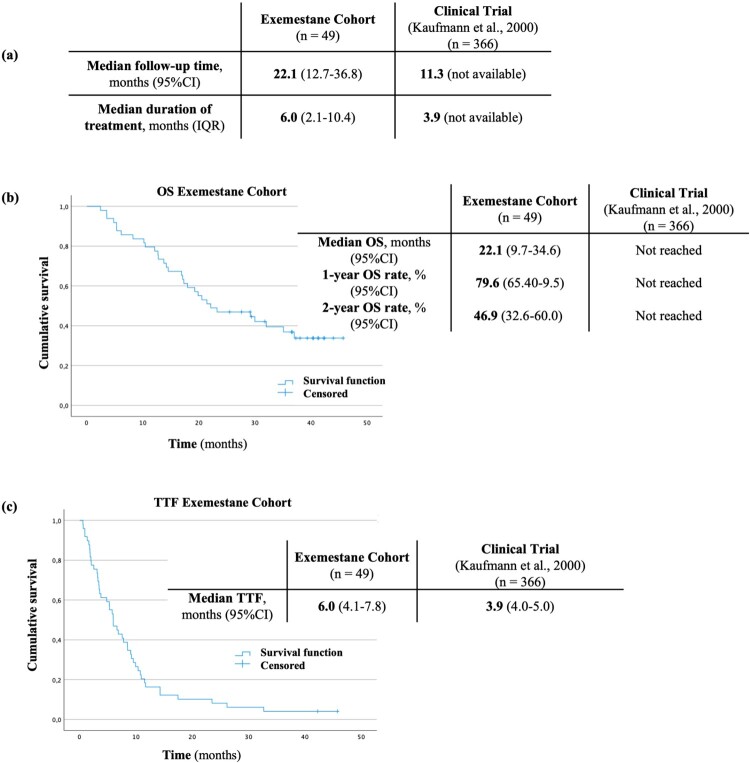
Median follow-up time (a), Kaplan–Meier estimate for OS (b) Kaplan–Meier estimate for TTF (c) in the exemestane cohort and the corresponding clinical trial.

All patients were treated with the recommended dosage, 25 mg/day. Median age at treatment initiation was 61 years (IQR: 51–76), compared to 65 years (IQR: 35–89) in the reference clinical trial. At treatment initiation, 98.0% of patients in this cohort and 100.0% of the patients in the trial had distant metastasis (Kaufmann et al., 2000). Almost all patients in our study had ECOG PS 0 or 1. In the trial, only median ECOG PS is presented (Table 3). Median follow-up in our study was 22.1 months (IQR: 12.7–36.8) and median treatment duration was 6.0 months (IQR 2.1–10.4) (Figure 3(A)). Median OS was 22.1 months (95%CI 9.7–34.6), and OS rates at 1- and 2-years were 79.6% (95%CI 65.4–88.5) and 46.9% (95%CI 32.6–60.0), respectively. A median TTF of 6.0 months (95%CI 4.1–7.8) was estimated. TTF was higher than that reported in the trial (3.9 months) (Kaufmann et al., 2000). It was not possible to compare the estimated OS for this cohort, as this was not achieved in the trial (Figure 3).

**Table 3.** Demographic and clinical characterisation of patients included in the exemestane cohort and in the respective clinical trial at treatment initiation.

		Exemestane Cohort (*n* = 49)	Clinical trial (Kaufmann et al., 2000) (*n* = 366)
Age at treatment initiation, years	Median (IQR 25–75)	61 (51–76)	65 (35–89)
	[Min.; Max.]	[37; 88]	Not available
Disease extent at treatment initiation, n (%)	IIIC	1 (2.0)	0 (0.0)
	IV	48 (98.0)	366 (100.0)
ECOG PS at treatment initiation, n (%)	0	21 (42.9)	Not available
	1	19 (38.8)	
	2	4 (8.2)	
	3	0 (0.0)	
	4	0 (0.0)	
	Unknown	5 (10.2)	

**IQR:** interquartile range; **ECOG PS:** Eastern Cooperative Oncology Group Performance Status.

### 3.3. Fulvestrant cohort

**Figure 4.**
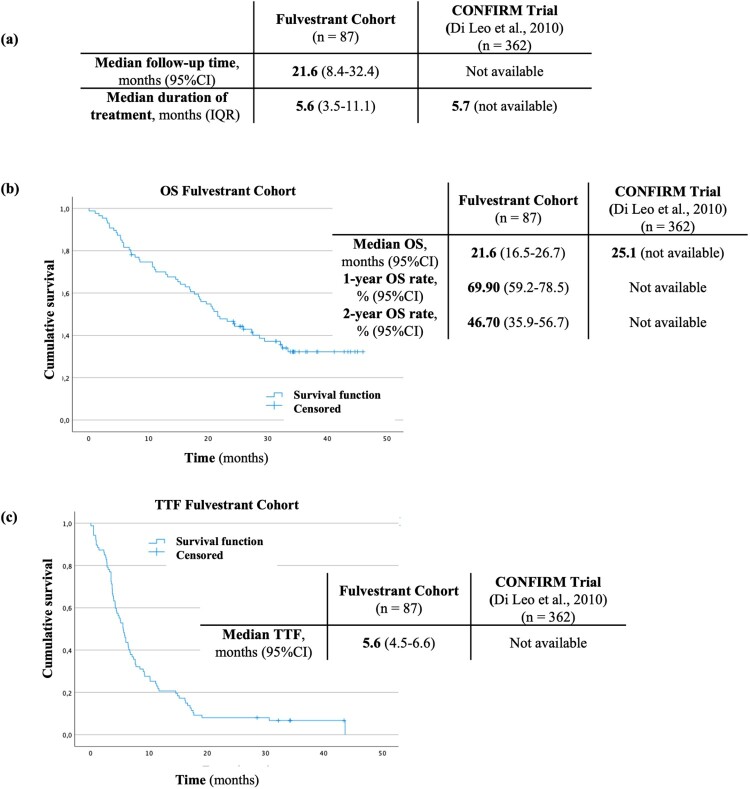
Median follow-up time (a), Kaplan–Meier estimate for OS (b), Kaplan–Meier estimate for TTF (c), in the fulvestrant cohort and the corresponding clinical trial.

All patients were treated with the recommended dosage, 500 mg/month. Patients who received fulvestrant had a median age of 63 years (IQR: 54–75) at treatment initiation, compared to 61 years (IQR not available) in the clinical trial. Almost all patients (96.6%) included in this cohort had distant metastasis by the time they started fulvestrant, identical to the patients of the trial (98.9%) (Di Leo et al., 2010). Approximately 70.0% of patients had an ECOG PS 0 or 1 at treatment initiation, not possible to compare as this variable was not described in the trial (Table 4). The median follow-up was 21.6 months (IQR: 8.4–32.4), and the median treatment duration was 5.6 months (IQR 3.6–11.1) (Figure 4(A)). Median OS was estimated at 21.6 months (95%CI 16.5–26.7), and median TTF was 5.6 months (95%CI 4.5–6.6). OS rates at 1- and 2-years were 69.9% (95%CI 59.2–78.5) and 46.7% (95%CI 35.9–56.7), respectively. Median OS was lower than that reported in the clinical trial (25.1 months) (Di Leo et al., 2010). TTF and 1- and 2-year OS rates were not described in the CONFIRM study, thus preventing comparison (Figure 4).

**Table 4.** Demographic and clinical characterisation of patients included in the fulvestrant cohort and in the respective clinical trial at treatment initiation.

	* *	Fulvestrant Cohort (*n* = 87)	CONFIRM trial (Di Leo et al., 2010) (*n* = 362)
Age at treatment initiation, years	Median (IQR 25–75)	63 (54–75)	61 (IQR not available)
	[Min.; Max.]	[38; 88]	Not available
Disease extent at treatment initiation, n (%)	IIIC	3 (3.5)	4 (1.1)
	IV	84 (96.6)	358 (98.9)
ECOG PS at treatment initiation, n (%)	0	35 (40.2)	Not available
	1	25 (28.7)	
	2	8 (9.2)	
	3	2 (2.3)	
	4	0 (0.0)	
	Unknown	17 (19.5)	

**IQR:** interquartile range; **ECOG PS:** Eastern Cooperative Oncology Group Performance Status.

### 3.4. Safety data

Treatment discontinuation occurred in 85.1%, 95.9% and 94.3% of patients in the letrozole, exemestane and fulvestrant cohorts, respectively. Our data showed that disease progression was the main reason for discontinuation in all three cohorts (Table 5). A minority of patients discontinued treatment due to AEs, which occurred in 6.8%, 12.8% and 1.2% of patients for letrozole, exemestane and fulvestrant, respectively. The proportion of patients discontinuing exemestane due to AEs in the trial was much lower (1.7%) (Kaufmann et al., 2000), whereas in fulvestrant, it was slightly higher (2.2%) (Di Leo et al., 2010). The letrozole clinical trial did not present this information. The most common AEs leading to discontinuation of both letrozole and exemestane was arthralgia, and the most common for fulvestrant was hypersensitivity (Table 6). None of the reference trials characterised AEs.

**Table 5.** Frequency of treatment discontinuation and respective reasons.

		Letrozole Cohort (*n* = 87)	Exemestane Cohort (*n* = 49)	Fulvestrant Cohort (*n* = 87)
Treatment discontinuation, n (%)	No	13 (14.9)	2 (4.1)	5 (5.8)
	Yes	74 (85.1)	47 (95.9)	82 (94.3)
Reason for discontinuation, n (%)	Disease progression	53 (71.6)	37 (78.7)	65 (79.3)
	Adverse events	5 (6.8)	6 (12.8)	1 (1.2)
	Refuse	0 (0.0)	0 (0.0)	1 (1.2)
	Death	14 (18.9)	3 (6.4)	4 (4.9)
	Other	2 (2.7)	1 (2.1)	11 (13.4)

**Table 6.** AEs that led to treatment discontinuation.

Adverse events leading to treatment discontinuation	Letrozole Cohort (*n* = 5)	Exemestane Cohort (*n* = 6)	Fulvestrant Cohort (*n* = 1)
Arthralgias, n (%)	3 (60.0)	3 (50.0)	0 (0.0)
Diarrhoea, n (%)	0 (0.0)	2 (33.3)	0 (0.0)
Cough, n (%)	1 (20.0)	0 (0.0)	0 (0.0)
Hepatotoxicity, n (%)	1 (20.0)	0 (0.0)	0 (0.0)
Palpitations, n (%)	0 (0.0)	1 (16.7)	0 (0.0)
Hypersensitivity, n (%)	0 (0.0)	0 (0.0)	1 (100.0)

## 4. Discussion

This study adds to the body of knowledge on the effectiveness of AIs and fulvestrant in the treatment of HR+/HER2- ABC, covering the last 2 years before CDK4/6i were conventionally used at IPOLFG. While the practical application of medicines in a real-world context tends to exhibit variability, leading to varied real-life cohorts, they may more closely reflect the characteristics of the typical population receiving treatment, thus offering a more precise portrayal of outcomes that may shape clinical expectations.

Our main findings provide further evidence of the benefits of monotherapy with AIs and fulvestrant in women with ABC, including an estimated median OS of 30.8, 22.1 and 21.6 months for letrozole, exemestane and fulvestrant, respectively. This information is very important in the context of health technology assessment as it may put in context real-world data published after CDK4/6i became the new standard of care (Alves da Costa et al., 2023; Cardoso Borges et al., 2022).

The indirect comparison of the estimated OS for the letrozole cohort with that reported in the trial suggests lower effectiveness in the real-world context (30.8 vs. 34.0 months). However, our study had a lower median follow-up time and median duration of treatment (Mouridsen et al., 2003). A different trend was observed for TTF, which was higher in the real-life setting (11.2 vs. 9.0 months). Worth stressing that in the trial, patients with central nervous system (CNS) metastasis, more than 50.0% lung metastasis, more than one-third of the liver involved or whose disease relapsed or recurred during adjuvant ET or within 12.0 months of completing such therapy were excluded, whereas in our study, there were no exclusions made based on the metastasis’s location. Additionally, having inflammatory breast cancer, other malignant diseases or some comorbidities (e.g. cardiac disease or diabetes mellitus) precluded enrolment in the trial. Finally, only patients with ECOG PS between 0 and 2 were included in the trial, which may be indicative of a worse functional status in the real-life setting. We have also compared our data with results from the letrozole + placebo group in the PALOMA-2 trial (Finn et al., 2016), since it included patients more recently treated and with similar baseline characteristics to those included in our study. We found that PFS was approximately 3.0 months higher than the TTF (14.5 vs. 11.2 months), which may result from PFS not including patients who discontinued treatment for reasons other than disease progression or death. Median OS presented in PALOMA-2 (Finn et al., 2022) was approximately 51.0 months, which is over twice that estimated for the letrozole cohort. However, the higher OS in the trial may be influenced by the subsequent use of CDK4/6i, which were not a standard practice in the Portuguese health system until 2021.

Median OS achieved in the exemestane cohort was 22.1 months, whereas in the trial it had not been reached. TTF was higher than that reported in the trial (6.0 vs. 3.9 months), which may result mainly from the way outcomes are measured in clinical trials, as opposed to real-world contexts. Our study had a higher median follow-up time and median duration of treatment than the clinical trial, which further values our study (Kaufmann et al., 2000). In this trial, patients with prior ET (except with tamoxifen), inflammatory carcinoma, rapid progressive disease, massive visceral disease, CNS metastasis and some comorbidities were excluded, which may cause an overestimation of OS. None of these exclusion criteria were considered in our study. We found no study reporting data from the use of exemestane in control groups of CDK4/6i trials, preventing further comparisons.

Median OS in the fulvestrant cohort and in the CONFIRM trial (Di Leo et al., 2010) may suggest greater efficacy than effectiveness (21.6 vs. 25.1 months). This difference of around 3.5 months can be due to the specific and temporal differences between studies. In the CONFIRM trial, exclusion criteria included extensive liver and/or lung metastasis, CNS metastasis and more than one chemotherapy or ET for advanced disease; none of which applied to our study. Median duration of treatment was similar in our study and in the reference trial and median follow-up time of patients was not reported in the trial. We further compared our data with those of the fulvestrant + placebo group in the PALOMA-3 trial (Turner et al., 2018), and found similar TTF and PFS (5.6 vs. 4.6 months) and higher OS in the trial (21.6 vs. 28.0). The differences in OS may also be related to the exclusion criteria applied in trials, as abovementioned.

Overall, OS was slightly higher in letrozole and fulvestrant clinical trials, suggesting a lower effectiveness of these drugs in a real-life context. This is not surprising as eligibility criteria of trials are stricter, and consequently, patients included may have better prognostic characteristics. Furthermore, in all clinical trials, only postmenopausal women were included. Our study included all women, regardless of menopausal status, which could influence the prognosis of the disease, as premenopausal status may be a factor of worse prognosis. Conversely, a higher TTF was identified in the letrozole and exemestane cohorts compared to the respective trials, however in the absence of significance this finding was disregarded.

The main reason for therapy discontinuation in all cohorts was disease progression. It is well known that ET is effective in HR+ tumours; however, in the long term, most patients develop hormone resistance, which may lead to disease progression (Hanker et al., 2020). Furthermore, a minority of patients discontinued treatment due to AEs. The proportion of patients discontinuing treatment as a result of AEs was 6.8%, 12.8% and 1.2% for letrozole, exemestane and fulvestrant, respectively. While some of these data were absent in reference trials, for those possible to compare, the considerable difference observed in the exemestane cohort should be stressed (12.8% vs. 1.7%). The safety profile of ET identified in this study is in accordance with the literature, suggesting that these medicines are well tolerated (Carson & Dear, 2019; Hanker et al., 2020). In addition, the lower rate of treatment discontinuation due to AEs in the fulvestrant cohort, when compared to the other cohorts, may presumably result from fulvestrant’s mechanism of action, which is specific to estrogen receptors in the mammary gland, thus not leading to systemic effects by blocking these receptors elsewhere (Carson & Dear, 2019). In our study, only AEs leading to treatment discontinuation were captured and therefore, other less severe AEs are not described.

Our study is valuable as it adds evidence about the real-world effectiveness of AIs and fulvestrant in the treatment of ABC. A strength of this study was the median follow-up, which was higher in the exemestane and fulvestrant cohorts than in their reference clinical trials. It was also possible to estimate the 1- and 2-year OS rates for all cohorts, which was not available in any of the trials. The fact that only 2.1% of patients were lost to follow-up was an additional strength, since it contributed to the validity of our results. Moreover, the review and update of eligible cases in the registry’s database ensured the extraction of data with adequate quality and greater exhaustiveness, thus contributing to the internal validity of the obtained results. Finally, this study generated information that will allow to carry out a comparative effectiveness study with the new HR+/HER2- ABC standard-of-care.

Despite its value, there are limitations worth acknowledging, namely the fact that reference trials used for indirect comparisons are ancient (having included women with diagnostic criteria and treatment patterns different from the current ones) and have a considerable amount of missing information, limiting comparisons. Nonetheless, the fact that they are ancient also reinforces the relevance of the current study in supplementing available information. The retrospective nature of the study impacts on the completeness and quality of data. For example, we cannot ensure that RECIST criteria had been followed. This was also an important reason to consider TTF (instead of PFS) as an outcome of interest. Although our study was conducted using data from a national cancer registry, the data extracted focused on a cohort of cases treated in the IPOLFG, limiting external validity. In addition, it could have been relevant to include men in this study, as they represented 5% of our population.

## 5. Conclusion

Our data suggest that the effectiveness of letrozole and fulvestrant can be inferior to efficacy. Although the observed difference was 3.2 months in the letrozole cohort and 3.5 months in the fulvestrant cohort, the clinical meaning of this difference can be uncertain and can be, in part, attributed to the stricter inclusion criteria in the trials. Additionally, we found a higher proportion of worse prognostic characteristics in patients treated in the real-world than in patients treated in a clinical trial environment.

These real-life data can be clinically relevant to evaluate the true benefit of these drugs in HR+/HER2- ABC.

## Authors contributions

*Conceptualization:* A.C.M. and F.A.C.; *methodology:* M.I.T. and F.A.C.; *investigation:* M.I.T.; *data extraction:* AM; *data processing:* M.I.T. and A.M.; *writing original draft:* M.I.T.; *review and editing:* A.M.; H.N.; A.C.M.; A.L. and F.A.C.; *supervision and formal analysis:* F.A.C. All authors have read and agreed to the published version of the manuscript.

## Data confidentiality

The authors declare having followed the protocols in use at their working centre regarding patients’ data publication.
